# Molecular basis for KDEL-mediated retrieval of escaped ER-resident proteins – SWEET talking the COPs

**DOI:** 10.1242/jcs.250100

**Published:** 2020-10-09

**Authors:** Simon Newstead, Francis Barr

**Affiliations:** Department of Biochemistry, University of Oxford, South Parks Rd, Oxford OX1 3QU, UK

**Keywords:** Membrane transport, Structural Biology, Trafficking receptors

## Abstract

Protein localisation in the cell is controlled through the function of trafficking receptors, which recognise specific signal sequences and direct cargo proteins to different locations. The KDEL receptor (KDELR) was one of the first intracellular trafficking receptors identified and plays an essential role in maintaining the integrity of the early secretory pathway. The receptor recognises variants of a canonical C-terminal Lys-Asp-Glu-Leu (KDEL) signal sequence on ER-resident proteins when these escape to the Golgi, and targets these proteins to COPI- coated vesicles for retrograde transport back to the ER. The empty receptor is then recycled from the ER back to the Golgi by COPII-coated vesicles. Crystal structures of the KDELR show that it is structurally related to the PQ-loop family of transporters that are found in both pro- and eukaryotes, and shuttle sugars, amino acids and vitamins across cellular membranes. Furthermore, analogous to PQ-loop transporters, the KDELR undergoes a pH-dependent and ligand-regulated conformational cycle. Here, we propose that the striking structural similarity between the KDELR and PQ-loop transporters reveals a connection between transport and trafficking in the cell, with important implications for understanding trafficking receptor evolution and function.

## Abstract (precis)

**Summary:** The structure of the KDEL receptor gives new insights into the close connection between trafficking and transport in the cell.

## Introduction

### Origins of the KDEL ER-retrieval system

A defining feature of eukaryotic cells are the internal membrane-bound organelles, characterized by their unique biochemical environments. These organelles are connected by a network of vesicle transport pathways, which function to maintain the unique composition of each organelle. A crucial aspect of these pathways is their selectivity for different proteins and lipids. For luminal proteins, this selectivity is created in part by the presence of specific transport signals that are recognised by cognate export or retrieval receptors and, in turn, interact with cytoplasmic vesicle coat protein complexes (COPs) (Bonifacino and Glick, 2004; Gomez-Navarro and Miller, 2016). The endoplasmic reticulum (ER) forms the starting point for the production of both membrane-bound and soluble secretory proteins, which are synthesised in this organelle before being transported to the Golgi. Besides protein export, cells retain millimolar levels of chaperones and other protein cofactors required for protein folding in the lumen of the ER, and discriminate them from newly synthesised secretory and membrane proteins leaving the ER (Ellgaard and Helenius, 2003). A seminal discovery in the field was the observation by Sean Munro and Hugh Pelham that many ER chaperones terminate with a Lys-Asp-Glu-Leu (KDEL) sequence and that this sequence is necessary for retention of the ER chaperone BiP (HSPA5) within the cell (Munro and Pelham, 1987). Furthermore, they found that a C-terminal KDEL signal is sufficient for ER retention when appended to lysozyme, a heterologous protein ordinarily not resident in the ER (Munro and Pelham, 1987). This second observation is crucial since it shows that other features of the retrieved protein are not essential for the retrieval process. Pelham used this observation as the basis for a budding yeast genetic screen, where the enzyme invertase was tagged with HDEL, the ER-retrieval signal found in this organism (Pelham, 1988). He reasoned that ER-retention-defective (*erd*) mutant cells unable to retain this invertase-HDEL fusion protein within the cell would secrete it into the external environment. This approach identified genes that encode components of the sorting system recognising C-terminal KDEL and HDEL sequences. Yeast ERD2, one of the genes identified in this way, encodes a 26 kDa membrane protein, predicted to have seven transmembrane domains that are required for retention of ER luminal chaperones bearing a C-terminal KDEL-type retrieval sequence (Semenza et al., 1990). Subsequent studies showed that this membrane protein confers specificity to the retrieval system and is the receptor responsible for directly binding KDEL signal sequences (Lewis and Pelham, 1990; Scheel and Pelham, 1998; Wilson et al., 1993). Although close homologues of the yeast *ERD2* gene are found throughout eukaryotes, the retrieval signal itself does show some variation. Whereas in mammals, the receptor binds to KDEL, RDEL and HDEL sequences, other organisms use alternative sequences, such as ADEL or DDEL signals (Lewis and Pelham, 1990; Pelham, 1992; Pidoux and Armstrong, 1992). Why these differences exist remains unclear. Nevertheless, the receptor has high sequence conservation between these diverse organisms, suggesting this retention system evolved early in the evolution of eukaryotic cells and operates through a conserved mechanism.

**Fig. 1.**
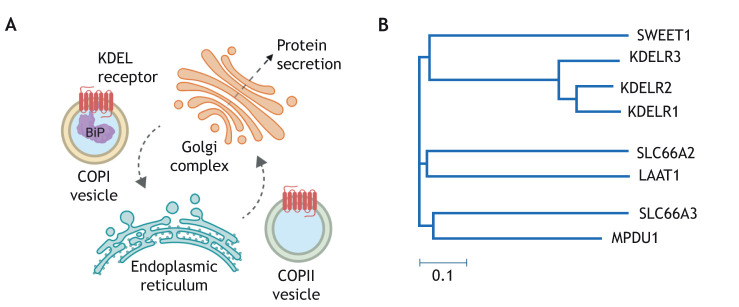
**The KDEL receptor functions to retrieve ER luminal proteins and is related to** SLC **transporters.** (A) The KDELR is located in the cis-Golgi, where it functions to selectively retrieve ER proteins that contain a carboxy-terminal Lys-Asp-Glu-Leu signal sequence in a pH-dependent process. In the Golgi, activation of the receptor leads to recruitment of COPI coatomer and retrograde trafficking back to the ER with the help of the ER chaperone BiP. Following the release of the cargo, the empty receptor recruits COPII coatomer for return to the Golgi. (B) The KDELR belongs to a functionally diverse group of heptahelical membrane proteins referred to as the ‘PQ-loop’ family. Within the PQ-loop family, the KDELR clusters together with the SWEET transporter that, in plants, is responsible for sugar transport.

As already mentioned, luminal ER chaperones are present at millimolar concentrations in the ER. However, the receptor is present at far lower concentrations within the cell, estimated to be ∼100-fold less in mouse and human cells (Itzhak et al., 2017, 2016). Early on, it was appreciated that KDEL retrieval is readily saturable and, therefore, must be a dynamic process where the receptor rapidly recycles between the ER and Golgi complex (Dean and Pelham, 1990). At steady-state, the KDEL receptor (KDELR) is mostly localised to the early or cis-Golgi, where it can efficiently capture escaped ER luminal proteins (Griffiths et al., 1994; Lewis and Pelham, 1992). Following retrieval to the ER, the bound cargo protein must rapidly dissociate, allowing the free receptor to return to the Golgi. During this cycle, chaperones, such as BiP and calreticulin, are known to remain bound to misfolded client proteins in the Golgi and to recycle with them back to the ER (Hammond and Helenius, 1994; Howe et al., 2009; Yamamoto et al., 2001). Thus, the retrieval signal of these ER-resident proteins is recognised by KDELRs either when bound to unfolded client proteins or in free form. This is likely to be of functional importance for cellular function, as it implies that the KDEL system retrieves both the escaped chaperone and any misfolded bound client protein to which it is bound. Binding of a protein with a C-terminal KDEL sequence to the receptor within the Golgi triggers incorporation of the receptor–protein complex into COPI vesicles (Gomez-Navarro and Miller, 2016). COPI vesicles return the receptor–cargo complex to the ER where the complex dissociates, and the cargo-free receptor is trafficked back to the Golgi complex via COPII vesicles (Gomez-Navarro and Miller, 2016) (Fig. 1A). The molecular basis for this differential regulation of COPI and COPII coat complexes has been unclear, although it must be regulated by ligand binding.

An essential aspect of this hypothesis is the requirement for KDEL-containing proteins to bind to the receptor in the Golgi followed by their rapid release into the environment of the ER. A simple explanation for this is the different luminal environments of these organelles, as the ER is of neutral pH with high Ca^2+^ levels and a reducing environment, whereas the Golgi has a mildly acidic pH with low Ca^2+^ levels and an oxidising environment (Kellokumpu, 2019). By using purified components, the pH – rather than Ca^2+^ levels or redox conditions – has been shown to be crucial for binding of KDEL ligands to the KDELR (Scheel and Pelham, 1998; Wilson et al., 1993). Although, interactions between the KDELR and its cargo are pH-sensitive (Wilson et al., 1993) with maximal binding at acidic pH values, the difference in proton concentration between the ER and Golgi is relatively small, i.e. in the range of ∼0.5–1 pH units (Wu et al., 2001, 2000). This raises a crucial question: how does this shallow pH gradient drive retrograde transport and concentrate chaperones to millimolar levels in the lumen of the ER? Until recently, the mechanistic basis for KDELR-mediated retrieval and the role of protons in ligand binding has remained stubbornly elusive. This is, in large part, due to the absence of structural insights into how this receptor engages different signal peptides and also the nature of the interactions that determine how the receptor discriminates between COPI or COPII complexes.

Recent crystal structures of the KDELR have now begun to address some of these questions (Bräuer et al., 2019). Captured in both peptide-free and KDEL-bound states, crystal structures of the chicken KDELR2 protein revealed a receptor that exhibits a high degree of both structural and mechanistic similarity to solute carrier (SLC) proteins, which are usually associated with transporting small molecules across membranes, rather than trafficking between different membranes. The ability of the KDELR to respond to changes in environmental pH also appears to display features common to proton-driven SLCs. In this Opinion article, we discuss the close structural homology between the KDELR and a family of bacterial and eukaryotic sugar transporters. Our comparison of the different structures highlights interesting structural and mechanistic similarities to SLC transporters that shuttle small molecules across the cell and organellular membranes. Furthermore, we provide important insights into the evolution of trafficking receptor function and the origins of selective protein transport mechanisms in eukaryotic cells.

## The diverse PQ-loop family – a minimal transporter

Phylogenetically, the KDELR belongs to a large and diverse family of integral membrane proteins, sometimes referred to as the Nodulin MtN3 family, which are characterised by the presence of a conserved Pro-Gln (PQ)-motif (Saudek, 2012). Originally, the PQ-motif was thought to reside in the loop regions connecting transmembrane helices 1 and 2 (TM1 and TM2) as well as TM5 and TM6, which resulted in the general name of PQ-loop proteins given to this family. In mammals, these include a family of putative sugar transporters called sugars will eventually be exported transporter (SWEETs) (Chen et al., 2010), the proton-coupled cystine transporter cystinosin (CTNS) (Kalatzis et al., 2004), cationic lysosomal amino acid transporters (LAATs/SLC66/PQLC2) (Jezegou et al., 2012) that are responsible for lysosomal homeostasis, as well as the mannose-P-dolichol utilisation defect 1 protein (MPDU1) that is required for normal utilisation of mannose-dolichol phosphate in glycosylation (Schenk et al., 2001) (Fig. 1B). The presence of transporters, trafficking receptors and glycosylation chaperones within one structural family is unusual. Although a range of different architectures have been reported for membrane proteins, these tend to cluster into defined functional families (Chang et al., 2004). Therefore, the clustering of several functionally distinct proteins into the PQ-loop family already marks this fold as being remarkably adaptable for a membrane protein.

**Fig. 2.**
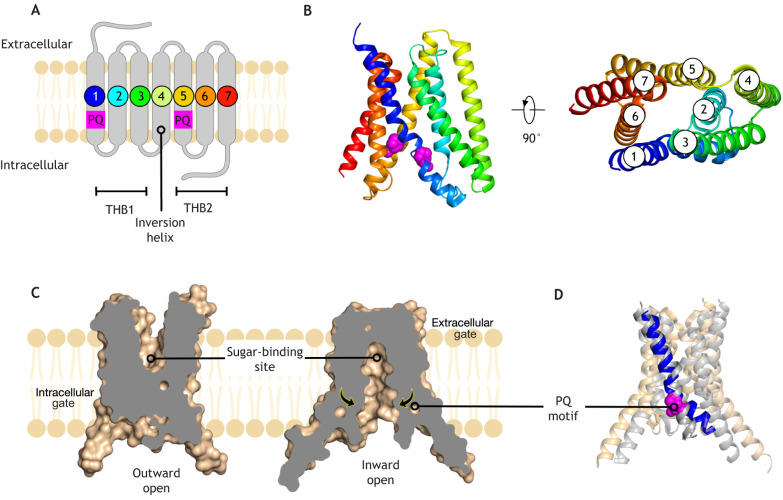
**Crystal structure of the SWEET sugar transporter.** (A) The eukaryotic SWEET transporter contains seven transmembrane alpha helices, which can be split into two three-helical bundles (THBs) (PDBe entry: 5ctg). The PQ sequence motif is located in the first helix of each THB, suggesting the full-length protein evolved by gene duplication. The fourth helix links the two THBs together and is often referred to as the inversion helix. (B) Crystal structure of the eukaryotic SWEET transporter coloured from N-terminus (blue) to C-terminus (red). The two PQ-motifs are highlighted and shown in space-filling representation (magenta). Right panel – view rotated 90°; helices are labelled. (C) Alternating access transport mechanism as revealed from crystal structures of the bacterial semiSWEET transporters (PDBe entries: 4×5n, 4×5m). The central sugar-binding site is labelled. Arrows indicate structural changes upon sugar binding. (D) The crystal structures of the outward- and inward-facing semiSWEET transporters (grey and beige, respectively) have been superimposed. Helix 1 has been coloured (blue) with the PQ-motif highlighted (magenta).

A significant breakthrough in our understanding of the PQ-loop family came with the identification of the SWEET family of sugar transporters, found in both bacteria, plants and mammals (Chen et al., 2010; Feng and Frommer, 2016). The SWEET family plays pivotal roles in mediating sugar transport in many organisms. In eukaryotes, these SWEET transporters contain seven TMs, whereas their bacterial homologues, the so-called semiSWEETs, contain only three TMs (Xuan et al., 2013). Crystal structures of bacterial semiSWEET transporters revealed that two triple helix bundles (THBs) associate to form a functional homodimer (Lee et al., 2015; Wang et al., 2014; Xu et al., 2014). Notably, the PQ-motif sits towards the end of TM1; we will return to this point later, when discussing the transport mechanism of these proteins. The structure of the first seven-TM-topology SWEET transporter, SWEET2b from the rice plant, further revealed that an inversion helix, TM4, connects the two THBs, enabling TMs1–3 to sit parallel to TMs5–7 within a single polypeptide chain (Tao et al., 2015) (Fig. 2A). The fusion of the two THBs – creating a new seven-TM transporter – enabled the PQ-loop fold to evolve new functions, as mutations could now occur separately in the two three-TM-bundles (Feng and Frommer, 2016). Indeed, in recent years, the fusion of simple three-TM, four-TM or five-TM bundles has emerged as a hallmark of SLC and receptor evolution (Forrest, 2015; Youkharibache et al., 2020). As is the case in the seven-TM PQ-loop transporter and the KDELR, the THBs are inverted 180° relative to one another within the membrane (Fig. 2B), and are known as inverted topology repeats or proto-domains. It is the coupling of structural movements in one repeat, with simultaneous but opposing movements in the other, that underlies the ability of SLCs to transport ions and molecules across the membrane (Forrest et al., 2011). However, to understand how the KDELR evolved to function as a trafficking receptor, it is first worth considering how the PQ-loop family functions as transporters.

## Mechanism of PQ-loop transporters

SLCs use an alternating access mechanism to shuttle ligands across the membrane. In this mechanism, a centrally located binding site alternates between either side of the membrane (Jardetzky, 1966). Alternating access requires SLC transporters to contain two regions of the protein that act as gates, enabling access to the binding site from one side of the membrane, while simultaneously closing off the binding site from the opposite side (Fig. 2C). During the transport cycle, the transporter must not adopt a conformation whereby both the intracellular and extracellular gates are open simultaneously. Such a conformation would result in dissipation of the membrane ion gradients and severely compromise cell integrity. Crystal structures of the bacterial semiSWEETs in both inward-facing (binding site facing the cytoplasm) and outward-facing (binding site facing the extracellular space) states have revealed that the PQ-motif plays a pivotal role in mediating the conformational changes during transport (Fig. 2C). (Latorraca et al., 2017; Lee et al., 2015; Xu et al., 2014). Proline (Pro) residues are well known to function as ‘helix breakers’ in membrane proteins, enabling helices to undergo more substantial conformational changes during transport and gating (Nilsson et al., 1998). In the PQ-loop family, the proline residue of the motif enables TM1 in each of the three-TM bundles to undergo a bending motion, which results in simultaneous closure of the intracellular gate and opening of the extracellular gate during transport (Fig. 2D). As semiSWEETs are homodimers, movement of one bundle is replicated in the other, resulting in a symmetrical movement that enables a bound sugar molecule to move across the bacterial inner membrane.

The situation in the rice SWEET2b transporter is slightly more complicated, as this protein has lost the glutamine side chain in the PQ-motif (Tao et al., 2015). Nevertheless, the existence of two conserved proline side chains at the equivalent location in TM1 and TM5 (Fig. 2A), demonstrates that similar helix dynamics occur in the absence of the glutamine. The structure of the rice transporter, thus, demonstrates that it is the proline residue in both TM1 and TM5, which enables PQ-loop proteins to undergo a symmetrical transport cycle. When comparing homologues of proteins from evolutionary distant species it is not uncommon to observe subtle changes in ancestral motifs (Yohannan et al., 2004). However, in the case of the KDELR, the first PQ-motif on TM1 is absent, resulting in a structurally asymmetric system (Bräuer et al., 2019; Saudek, 2012). As discussed below, this asymmetry – and the loss of the first PQ-motif – was probably a key feature in the evolution of the receptor.

## The KDEL receptor – a receptor disguised in transporter clothing

**Fig. 3.**
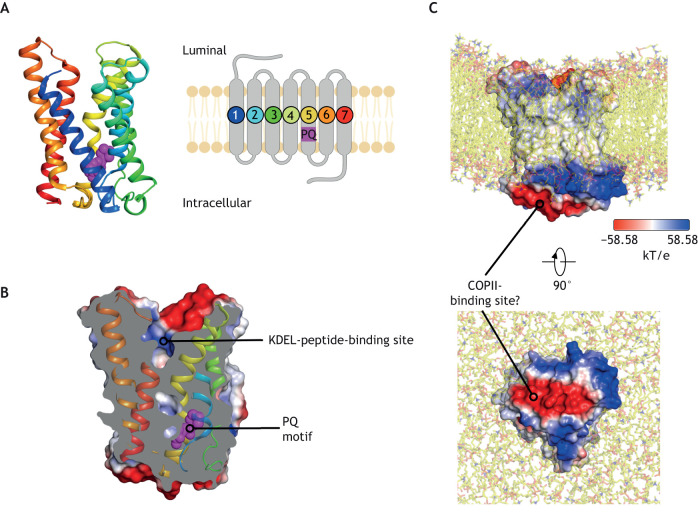
**Crystal structure of the KDEL receptor.** (A) Cartoon representation of the KDELR, coloured from the N-terminus (blue) to the C-terminus (red) (PDBe entry :6i6b). The PQ-motif in helix five is shown in sphere representation (magenta). The topology diagram on the right shows helix labels and location of the PQ-motif. (B) Space-filling representation of the KDELR showing a slice through the protein volume. The electrostatic surface of the protein is shown, with helices coloured as defined in A. The location of the KDEL-peptide-binding site and PQ-motif are indicated. (C) Electrostatic representation of the KDELR embedded in a membrane bilayer by using MemProtMD (Stansfeld et al., 2015). The cytoplasmic surface of the receptor projects away from the membrane surface, providing an ideal site to bind COPI or COPII complexes. The electrostatic potential (kT/e) has been mapped onto the surface of the protein, showing areas of negative (red) and positive (blue) charge.

The crystal structure of the KDELR shows that the overall topology is identical to that observed for the eukaryotic SWEET transporter (Bräuer et al., 2019), with two THBs connected by an inversion helix (Fig. 3A). A sizeable polar cavity extending from the luminal side of the Golgi membrane towards the centre of the receptor is the site of signal peptide binding (Fig. 3B). Following transporter nomenclature, the receptor adopts an outward open state, consistent with the role of the receptor in adopting a state that needs to sample the luminal environment. The receptor contains only a single PQ-motif, located on the cytoplasmic side of TM5 and, as noted above, the presumptive PQ-motif within TM1 has been lost (Bräuer et al., 2019). As discussed below, removal of the PQ-motif in TM1 was probably essential in the evolution of receptor functionality.

The cytoplasmic face of the receptor is thought to sit proud of the membrane, facilitating binding to COPI or COPII complexes (Bräuer et al., 2019). We can infer this from the noticeably thin hydrophobic surface of the receptor, which measures only 33 Å at its widest point. Thin hydrophobic surfaces due to shorter transmembrane domains are also observed in other ER and Golgi-resident membrane proteins, including Golgi nucleotide sugar transporters (Parker and Newstead, 2017; Sharpe et al., 2010), in agreement with these organelles having thinner membrane bilayers (Bretscher and Munro, 1993). Consistent with these ideas, the receptor adopts an asymmetric position in a self-assembled phospholipid bilayer and can be seen to project away from the cytoplasmic side of the membrane (Fig. 3C).

The electrostatic surface on the cytoplasmic side of the receptor is noticeably charged, with a prominent central band of negative charge running along the centre of the cytosolic face. The negative charge is contributed by several conserved acidic residues (Bräuer et al., 2019). Mutagenesis studies implicate several residues within the cytoplasmic portion of human KDELR that result in complete retention of the receptor in the ER (Townsley et al., 1993), suggesting this forms part of an acidic COPII-recognition motif (Barlowe, 2003).

## KDEL binding results in activation of the COPI-interaction site in the KDEL receptor

**Fig. 4.**
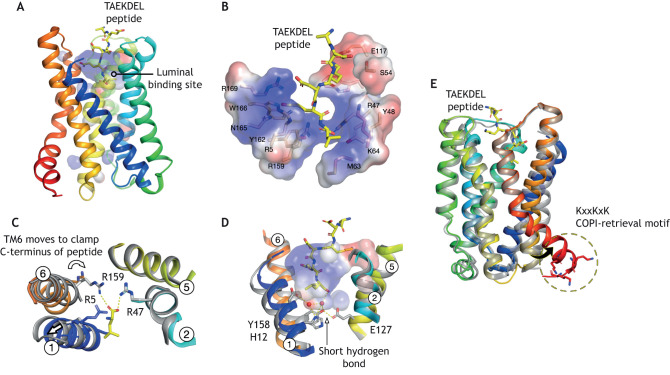
**Mechanism of receptor activation.** (A) Crystal structure of the KDELR bound to the TAEKDEL peptide (PDBe entry: 6i6h). The electrostatic surface of the peptide-binding site is shown. (B). Close up view of the peptide-binding site shown in A. The TAEKDEL peptide is shown in sticks (yellow), with key binding site side chains highlighted (wheat). (C) Top-down view of the peptide-binding site, showing the structural changes accompanying peptide binding. (D) Close-up view of the peptide-binding site shown in A, displaying the H_2_O molecules bound at the base of the pocket. (E) Comparison of the inactive (grey) and activated (coloured) receptor. The key structural change of the cytoplasmic side of the receptor is highlighted.

The mechanism through which peptide binding activates the KDELR was first explored in extensive mutagenesis studies, which identified several crucial residues (Townsley et al., 1993). We were able to build on that work and crystallise the receptor bound to a TAEKDEL peptide . Assisted by this new structural information, we could begin to describe the mechanism by which the receptor signals across the membrane to recruit COPI and initiate retrograde transport (Bräuer et al., 2019). Recognition of the TAEKDEL peptide is achieved predominantly through electrostatic interactions, with the positive amine group of the lysine (Lys) residue accommodated in a negative pocket, and the three carboxyl groups from the aspartate, glutamate and C-terminus of the peptide being accommodated in positively charged pockets (Bräuer et al., 2019) (Fig. 4A and B). This form of peptide recognition is noticeably different to that observed in peptide-activated G protein-coupled receptors, suggesting a different mechanism for ligand recognition (Cao et al., 2018). Of particular note in the KDELR are two salt-bridge interactions between the C-terminus of the peptide and residues Arg47 (TM2) and Arg159 (TM6) (Fig. 4C). This interaction results from rotation of TM6, causing Arg159 to move into the binding site and engage the C-terminus of the peptide. Once engaged, the receptor holds TM6 in place through the formation of a short hydrogen bond (SHB) between Glu127 on TM5 and Tyr158 on TM6 (Fig. 4D), preventing release of the peptide (Bräuer et al., 2019).

The formation of SHBs is unusual in non-enzymatic systems, as these are usually attributed to transition-state stabilisation (Cleland and Kreevoy, 1994). Consistent with this role, SHBs are much stronger than traditional hydrogen bonds and can contribute up to 10 kcal/mol in free energy to the stabilisation of a molecule (Cleland and Kreevoy, 1994). However, SHBs do not form spontaneously and usually require an input of energy (Cleland and Kreevoy, 1994). In the KDELR, it appears this energy is provided through the protonation of the nearby His12 residue on TM1. As both Tyr158 and His12 contain delocalised electron systems, protonation of His12 might stabilise the SHB interaction. Although yet to be experimentally proven, it is appealing to make this assumption based on the proximity of His12 and Tyr158; moreover, the favourable pKa of ∼6.5 for His makes it ideal for fast proton-transfer events at physiological pH. It is also interesting that, in the peptide-bound state, His12 interacts with the C-terminal of the peptide through a H_2_O-mediated hydrogen bond that may function to relay protons onto the side chain (Bräuer et al., 2019) (Fig. 4D). H_2_O molecules are known to function as proton-relay systems in proton-coupled transporters (Parker et al., 2017). Following peptide-binding in the acidic environment of the Golgi complex, H_2_O would be trapped at the base of the binding pocket, resulting in protonation of His12. This proposed mechanism neatly links receptor protonation to high-affinity peptide binding, and is currently being explored in our laboratory.

The movement and stabilisation of TM6 following peptide-binding on the luminal side of the membrane requires TM7 to change position, moving away from TM5 in a hinge-like movement on the cytoplasmic side (Bräuer et al., 2019) (Fig. 4E). This structural change is very similar to that observed in semiSWEET transporters when they transition between outward- and inward-facing states (Latorraca et al., 2017; Lee et al., 2015). A further important structural change occurs at the C-terminal end of TM7, which is likely to be disordered in the receptor in its unbound (apo) state, i.e. without its peptide ligand. Following peptide binding, the disordered C-terminus extends back into the receptor, resulting in the ordering of three conserved lysine side chains Lys201, Lys204 and Lys206 that now project out into the cytoplasm (Bräuer et al., 2019). The partially exposed Lys residue cluster is reminiscent of the KKxx and KxKxx di-lysine motifs (x representing any amino acid), which are essential in COPI-dependent Golgi to ER transport (Jackson et al., 2012; Letourneur et al., 1994). The exposure of these lysine side chains is likely to be the primary signalling mechanism in order to recruit COPI to the KDELR–cargo complex. Movement of TM6 and TM7 also results in a significant change in the electrostatic surface at the cytoplasmic end of the receptor. This movement divides the negatively charged band observed in the receptor in the apo state (Bräuer et al., 2019) (Fig. 3C). If this negative charge is the COPII- recognition motif, activation of the receptor would couple exposure of the COPI-binding site to the dissolution of the COPII site. This mutually exclusive signalling mechanism would be a very satisfying and mechanistically simple explanation for the selective recruitment of different coat complexs by the KDELR.

## Does the KDEL receptor use a modified transporter mechanism?

The KDELR selectively captures escaped luminal ER proteins in the Golgi complex and returns them to the ER (Dean and Pelham, 1990; Lewis and Pelham, 1990; Semenza et al., 1990). To carry out this function, the receptor must selectively recognise and bind the KDEL signal peptide in the Golgi and release it in the ER. The ability to signal across the membrane must be coupled to ligand recognition in order to recruit the COPI coatomer complex that drives retrograde trafficking. Interestingly, several similarities exist between these requirements and the mechanism of SLC transporters, which may explain why a transport system was repurposed for a Golgi–ER retrieval system. The first is the ability to modulate ligand binding. Transporters selectively recognise ligands on one side of the membrane and release them on the other (Forrest et al., 2011). The free energy contained within ion gradients is often used to concentrate molecules across membranes and drive transport in one direction. In mammalian cells, the change in luminal pH is an important characteristic of internal organelle biochemistry, making proton-coupled systems particularly useful to mediate transport processes within the cell (Nicholls and Ferguson, 2013).

Similarly, the KDELR system employs the pH difference between the Golgi complex and the ER to drive the direction of trafficking of KDEL-bearing cargo proteins. In the Golgi, the KDELR becomes protonated on His12, priming the receptor (Bräuer et al., 2019). Following binding of a KDEL-containing cargo protein, the receptor locks the KDEL peptide in place through the formation of a SHB (Bräuer et al., 2019). In this activated state, the receptor is predicted to bind to COPI on the cytoplasmic side of the membrane through ordering of the KxxKxK motif on TM7 (Jackson et al., 2012). After being returned to the ER, the change in pH results in deprotonation of His12 and destabilisation of the SHB (Wu et al., 2020 preprint). The receptor will then relax back to its apo state and release the peptide. As the receptor is no longer protonated, further interactions of KDEL-bearing proteins do not result in the formation of stable interactions, even though these are present at millimolar concentration in the ER. The C-terminal end of TM7 can now pack against the receptor, reforming the COPII-binding site and initiating the return of the receptor to the Golgi.

Interestingly, the role of protons in this mechanism is not wholly dissimilar to that found in *Escherichia coli* lactose permease (LacY), possibly the best-studied proton-coupled transporter in biology. In LacY, protonation precedes lactose binding (Smirnova et al., 2012), and it is proton binding and release that enables the transporter to alternate between inward- and outward-facing states during transport (Jiang et al., 2020). Similarly, in the KDELR, proton binding and release also facilitate the structural transition between active and inactive states of the receptor.

## The KDEL receptor – an evolutionary link between transport and signalling

The evolution of receptor function in biology is an interesting area of research. A plausible hypothesis for the origin of receptor function is that receptors evolved from solute transporters, as these molecules have many features essential for a membrane receptor, such as a high-affinity binding site and the ability to couple ligand-binding on one side of the membrane to conformational changes on the other. Indeed, several SLC systems still function as both transporters and signal transduction receptors, the so-called transceptors (Hundal and Taylor, 2009). Interestingly most transceptors identified to date are linked to nutrient sensing, either nitrogen in plants (Ho et al., 2009) or amino acids in fungi and mammals (Kriel et al., 2011). In humans, several transceptors have been linked to metabolic dysregulation during cancer, and shown to traffic between the plasma membrane and the lysosome during cell growth and division (Heublein et al., 2010). The structural similarity between the KDELR and PQ-loop transporters suggests that a similar evolutionary path has been followed. The ability of the receptor to respond to pH changes implies a link to proton-coupled transporters, whereas recognition of the free carboxyl group at the base of the luminal binding pocket displays remarkable similarity to amino acid transporters (Jungnickel et al., 2018; Penmatsa and Gouaux, 2014). We speculate then, that the KDELR evolved from an ancestral proton-coupled amino acid transporter that was repurposed to function as a trafficking receptor with, potentially, more-complex signalling roles in regulating ER–Golgi dynamics. Several studies have reported that binding of ER chaperones to the KDELR can trigger a Gq-dependent activation of the Src kinase cascade, resulting in increased intra-Golgi trafficking (Giannotta et al., 2012; Pulvirenti et al., 2008). At present, the structural features required for signalling within the Golgi remain unclear and require further investigation to uncover the mechanism of G-protein activation.

**Fig. 5.**
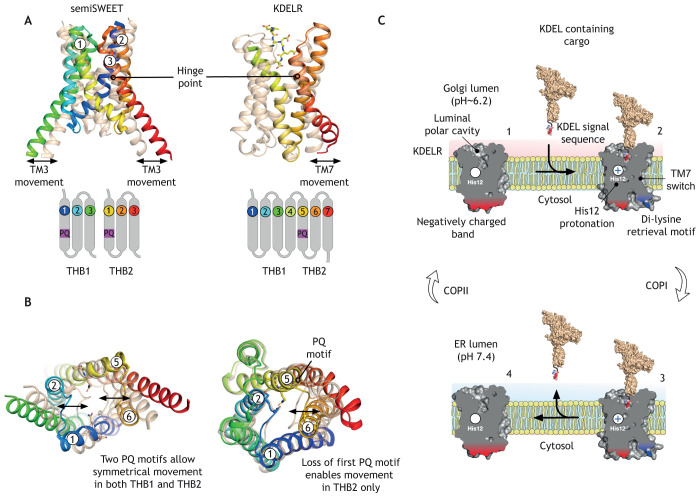
**The KDELR and SWEET transporters share similar structural and mechanistic framework.** (A) Crystal structures of the semiSWEET (PDBe entries:4×5n, 4×5m) and KDELR (PDBe entries: 6i6b, 6i6h) are shown overlaid. In the symmetrical semiSWEET proteins, transport results in movement in both three-helix bundles (THBs). This contrasts with the KDELR, where activation results in movement in only the second THB, which contains the PQ-motif. (B) View of structures shown in A rotated 90°. (C). The retrieval cycle of the KDELR can be viewed as a one-half cycle of a transporter. In the Golgi, the receptor is protonated at His12, creating a high-affinity binding site for the KDEL-retrieval sequence (1). Binding to KDEL-tagged cargo triggers the formation of a stable receptor–cargo complex and incorporation into COPI vesicles (2). Following trafficking to the ER (3), the receptor–cargo complex disassociates following release of the proton in the neutral pH environment (4), effectively reversing the previous step. Finally, the deprotonated receptor returns to the Golgi via the COPII system to complete the cycle (1). Panel C was reproduced from Bräuer et al., 2019.

How then does a receptor evolve from a transporter? Similarities can be found between the structural changes of KDELRs following activation and the alternating access transport in PQ-loop proteins. During the transport cycle of the semiSWEET proteins, theTM3 in each THB undergoes a similar hinge-like movement to that observed in TM7 of the KDELR (Latorraca et al., 2017; Lee et al., 2015; Bräuer et al., 2019) (Fig. 5A). However, whereas in the semiSWEET transporter we observe movement in all three helices within each THB, in the KDELR we see movement only in TM6 and TM7 of THB2. Conspicuously, TM5 – which contains the PQ-motif in the KDELR – does not undergo any significant movement between apo and peptide-bound (holo) state of the receptor. We can explain this through the observation that the symmetry-related PQ-motif within THB1 of the KDELR has been lost and replaced by bulky isoleucine side chains. The loss of the PQ-motif in TM1 results in a far more stable helix. The rigidity of TM1 is further supported by the presence of the peptide, which physically holds the luminal side of the receptor open following binding (Fig. 4D). The increased stability of TM1 increases the stability of TM2 and TM3. Indeed, we can see that, upon peptide binding, no significant structural changes occur in THB1 at all (Fig. 4E). The inability of TM1 to undergo a hinge-like movement explains why a reciprocal movement is not observed in TM5 of the KDELR. Here, the rigidity of TM1 results in the inability to break the interaction between the PQ-motif in TM5 and the loop connecting TM1 and TM2 (Fig. 5B). Thus, even though the KDELR contains a PQ-motif in THB2, it is functionally redundant without its opposing partner in the THB1 (Bräuer et al., 2019). This contrasts with the situation in the semiSWEET transporters, where the movement of each THB occurs simultaneously, breaking the interaction network between TM1 and TM2, and TM5 and TM6, which form the extracellular and intracellular gates that mediate transport (Fig. 2C) (Latorraca et al., 2017; Lee et al., 2015).

In practice, the KDELR can be thought of as undergoing one half of a full transporter cycle (Fig. 5C). Instead of cycling between inward- and outward-facing states like a transporter, the receptor undergoes only the first half of the transport cycle due to its ability to only move helices in THB2 of the structure. Essentially, the receptor undergoes one forward half of a transport cycle in the Golgi where the acidic pH of ∼6.2 drives formation of the KDEL-bound cargo complex, and the reverse half-cycle in the ER where deprotonation causes the cargo to disassociate.

## Conclusions and perspectives – KDEL receptor neither boring nor broken

The crystal structure of the KDELR has revealed several new and exciting links between trafficking receptors and the SLC family of membrane proteins. Although often seen as two distinct disciplines in cell biology, a growing body of evidence reveals links between transporters and receptors (Hundal and Taylor, 2009; Kriel et al., 2011). Transporters trafficking through the endocytic pathway play a central role in the regulation of amino acid metabolism in mammalian cells through the mTOR signalling pathway (Saxton and Sabatini, 2017). However, despite this growing understanding, many important questions remain. We have discussed here the similarities between mechanisms for KDELR function and proton-coupled transporters. However, it is premature to conclude that these are general principles that apply to other trafficking receptors. Investigation of other trafficking receptors in the cell and, in particular, whether they share similar features with the wider family of SLCs is required to answer this question.

Concerning the KDELR, there is also much that still needs to be understood (Pfeffer, 2007). Perhaps one of the more enigmatic questions concerns the role of lipids in regulating trafficking events within the secretory pathway. The recent discovery that short-chain lipids regulate nucleotide sugar transport in the Golgi complex (Parker and Newstead, 2017) demonstrates the important, and yet poorly understood, role of lipids in regulating SLCs in eukaryotic cells (Parker et al., 2019). Coupled with the well-established role of membrane thickness in the sorting of membrane proteins within the secretory pathway (Sharpe et al., 2010), it seems probable that lipids will emerge as significant regulators of trafficking receptors – as they have for transporters and channels (Gupta et al., 2017).

How trafficking receptors discriminate between different sorting signals in the crowded luminal environment of the secretory pathway is also an area of intense investigation (Gomez-Navarro and Miller, 2016). The linear signal of the KDELR is conceptually easier to understand compared to forward trafficking systems, which must discriminate between proteins in different stages of folding and glycosylation. Nevertheless, questions remain concerning how the KDELR distinguishes between KDEL, HDEL and RDEL ligands in mammalian cells. Here, the current structures alone are insufficient, as the residues that interact with the KDEL peptide are conserved in the budding yeast HDEL receptor. Our structural analysis indicates that key contacts between the KDEL sequence and the receptor are made to the C-terminus of the peptide, and perturbation of these interactions disrupts binding and retrieval function in cells (Bräuer et al., 2019). Munro and Pelham's original study used the last six amino acids of BiP, i.e. AEKDEL, as a retrieval signal (Munro and Pelham, 1987), and more recent work suggested that both the 5 and 6 positions are important determinants for recognition (Alanen et al., 2011). Again, this requires further investigation, although the current structures indicate that these residues are not crucial for signal recognition. Further questions surround the existence of three KDELRs in mammalian cells (Trychta et al., 2018). KDELR2 appears to be the predominant form in a variety of human cell lines and tissues and, together with KDELR3, is upregulated under stress conditions (Raykhel et al., 2007). Here, the availability of subtype-specific nanobodies that discriminate between the different isoforms will be invaluable, as will the availability of biochemical assays to measure the kinetics of cargo-receptor interactions. Finally, the role of receptor dynamics in cargo binding, and mechanism of COPI and COPII recruitment can now be studied in detail.

It seems fitting to close this review with the words of the late Professor Ron Kaback (UCLA) (Carrasco, 2020), a pioneer in the field of membrane transport biochemistry, “…the most interesting and important membrane proteins are transporters because they can transduce energy into work in the form of a concentration gradient. In contrast, channels are boring holes, which merely allow ions and such to flow down their activity gradients. Their only interesting property is gating, because gating is similar to transport. Receptors are obviously broken transporters that bind ligands but forgot how to transport them across the membrane”. The KDELR, of course, is neither boring nor broken but is most definitely interesting and important.
